# Concurrent Radiosurgery and Systemic Therapies for Melanoma Brain Metastases: A Systematic Review

**DOI:** 10.7759/cureus.6147

**Published:** 2019-11-13

**Authors:** Bradley D Weaver, James R Goodman, Randy Jensen

**Affiliations:** 1 Oncology, University of Utah, Salt Lake City, USA; 2 Anesthesiology, Oregon Health & Science University, Portland, USA; 3 Neurosurgery, University of Utah, Salt Lake City, USA

**Keywords:** melanoma, stereotactic radiosurgery, targeted therapy, immunotherapy, brain metastases

## Abstract

Intracranial metastatic melanoma is a major challenge for neuro-oncological teams. Historically, treatment has focused on surgical or radiosurgical treatment of appropriate lesions, mostly for palliative purposes. Immunotherapies and other targeted therapies (BRAF/mitogen-activated protein kinase kinase inhibitors (BRAFi/MEKi)) are mainstays of advanced melanoma therapy, yet the optimal timing and synergistic properties of concurrent combinations of these systemic therapies and stereotactic radiosurgery (SRS) are poorly understood. We performed a systematic review of the MEDLINE and Scopus databases focused on outcomes after therapy using SRS and either immunotherapies or targeted therapies in an effort to define the optimal timing. We defined concurrent therapy as SRS within three months of treatment with any systemic therapy. End points included local control, distant control, overall survival, and toxicities. We identified five retrospective cohort studies from the literature. These studies found that concurrent SRS plus immunotherapy or BRAFi/MEKi is well tolerated by most patients and generally improved local control, distant control, and overall survival. Importantly, no significant increases in toxicities were noted with concurrent therapy. Combining concurrent SRS with immunotherapy or BRAFi/MEKi may offer important advances for patients with intracranial metastatic melanoma. To address interstudy heterogeneity, we propose reporting two major time intervals defining “concurrent treatment”: concurrent-SRS (≤4 weeks) and peri-SRS (≤3 months). Future large-scale, prospective trials considering truly concurrent SRS therapies with systemic therapies are desperately needed.

## Introduction and background

Intracranial metastatic melanoma is a devastating and common occurrence in patients with advanced melanoma. As of 2011, more than 40% of patients with metastatic melanoma experienced brain metastasis, and this number is increasing [1-2]. Stereotactic radiosurgery (SRS) is a safe and effective modality for treating many types of primary and metastatic brain tumors and is commonly used for the treatment of melanoma brain metastasis. BRAF-V600E is the most common activating mutation found in melanoma. After the molecular diagnosis is established, targeted therapies (TTs) such as BRAF/mitogen-activated protein (MAP) kinase kinase (MEK) inhibitors (BRAFi/MEKi), which block the activated MAP-kinase cascade are employed [3]. Immunotherapeutics (IMTs) such as ipilimumab (anti-CTLA4 [cytotoxic T-lymphocyte associated protein 4] therapy) have yielded improved overall survival from metastatic melanoma (two large, phase III trials), and along with nivolumab/pembrolizumab (anti-PD1 [programmed cell death protein 1] therapy), comprise the cornerstone of current melanoma immunotherapy [4-5]. Recently, phase II trials have begun investigating the effectiveness of IMTs with and without SRS [NCT02085070; NCT02374242; NCT02460068; NCT02320058]. Importantly, the optimal timing of combination systemic therapy and SRS is yet to be defined, particularly for IMTs, and preclinical studies suggest that concurrent therapy may be superior to staggered drug and SRS administration [6]. We undertook a systematic review of studies involving a window of concurrent systemic therapy within three months of SRS treatment, defined as administration of IMT or TT within three months of SRS, in an effort to better define the optimal timing.

## Review

Database review

Two separate reviewers performed Preferred Reporting Items for Systematic Reviews and Meta-analyses (PRISMA)-based systematic reviews of both Scopus and MEDLINE databases (October 2018) using “stereotactic radiosurgery” and “melanoma” as keywords. Articles were included if they examined the treatment of intracranial metastatic melanoma with SRS and BRAFi/MEKi inhibitors or immunotherapeutic checkpoint inhibitors (i.e., anti-PD1 and anti-CTLA4 monoclonal antibodies). Studies were included if they reported ≤30% of patients previously, or concurrently, treated with whole-brain radiotherapy. Critically, studies were included only if they described concurrent combinations of SRS and systemic therapies (i.e., systemic therapy within a three-month window before or after SRS treatment) (Table 1). Endpoints of interest included overall survival, local control, distant control, and treatment toxicities.

**Figure 1:**
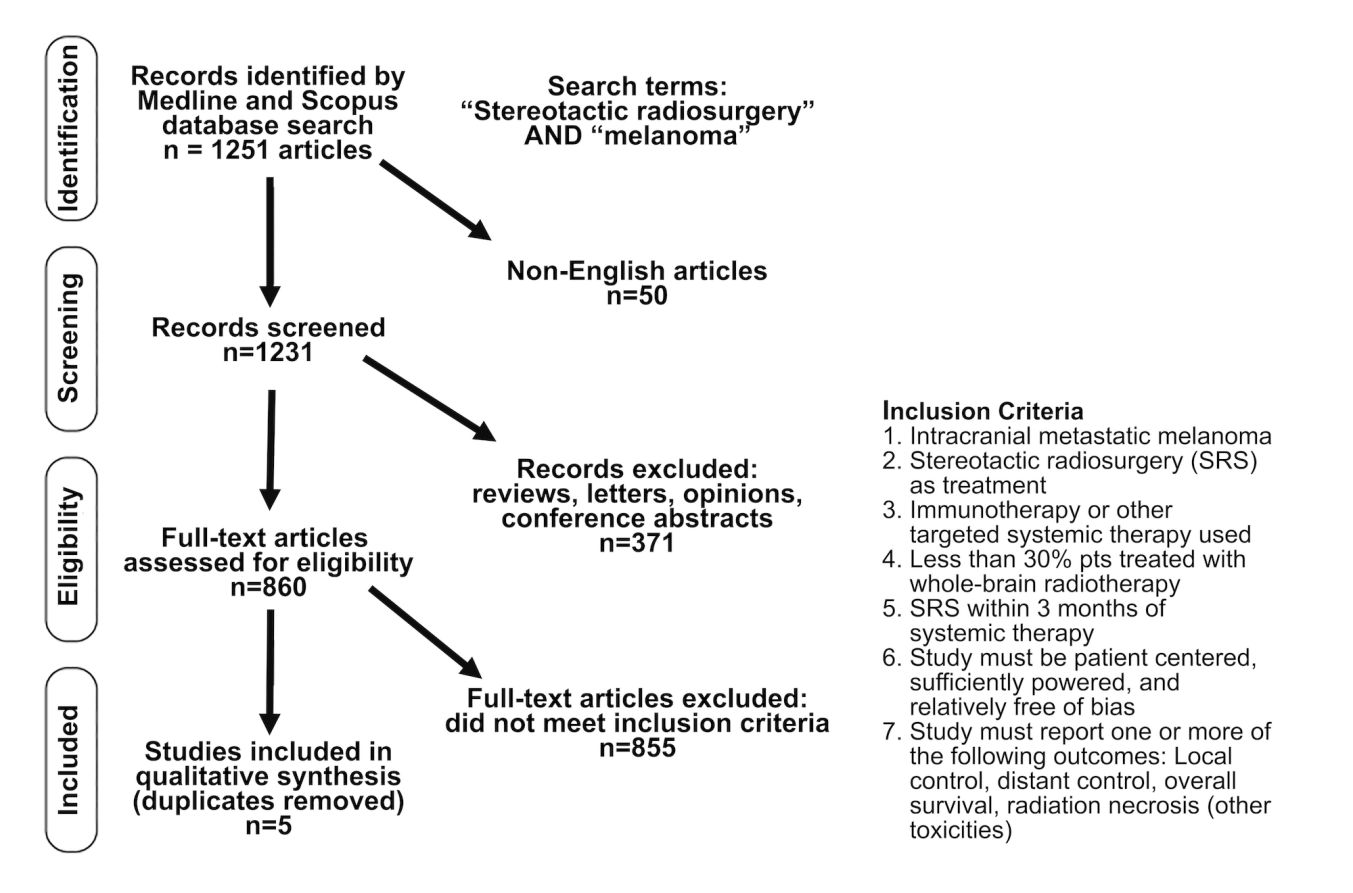
Diagram of PRISMA workflow representing search strategy, results, and inclusion criteria

After a systematic review of the literature, five articles were included for qualitative analysis (Figure 1). Hazard ratios (HRs) reported were transformed from failure to control, if necessary, using 1/HR. If a study presented both univariate and multivariate analyses of an outcome measure, the multivariate analysis was included in our qualitative assessment.

Studies identified

The five retrospective cohort studies that met our inclusion criteria all provided only low-quality evidence with heterogeneous outcomes (Table 1; GRADE (Grading of Recommendations Assessment, Development, and Evaluation) criteria); therefore, a meta-analysis of the data was not possible [7-11]. A major source of heterogeneity in all studies of SRS and systemic therapies is the definition of "concurrent" therapy. To address this issue, we propose assessment during two key intervals: a four-week interval (concurrent therapy) and a three-month window (peri-SRS therapy).

**Table 1:** Details of the retrospective cohort studies included in this review WBRT, whole-brain radiation therapy; SRS, stereotactic radiosurgery; PD1, programmed cell death protein 1; CTLA4, cytotoxic T-lymphocyte-associated protein 4; LINAC, linear accelerator; BRAFi, BRAF inhibitor; MEKi, MEK inhibitor [7-11]

Article	Number of patients	Total no. of brain mets	Type of radiosurgery	Type(s) of targeted and immunotherapies	Concurrent treatment definition	Patients (% of total) who had WBRT	Endpoints measured	Statistics used	GRADE quality and bias assessment
Acharya et al. (2017)	72	233	Single-fraction SRS Leksell Gamma Knife	Anti-PD1/anti-CTLA4 = nivolumab/ pembrolizumab, ipilimumab; BRAFi/MEKi = dabrafenib/ trametinib, vemurafenib	3 months	9.7	Distant brain failure, local failure, overall survival, neurotoxicity	Fisher's exact test and Wilcoxon rank-sum; Kaplan Meier and Cox proportional hazards regression model for hazard ratios	LOW: small, retrospective cohort study. No downgrade required.
Ahmed et al. (2016)	96	314	Single-fraction BrainLab Novalis Classic LINAC	Anti-PD1/anti-CTLA4 = nivolumab/ pembrolizumab, ipilimumab; BRAFi/MEKi = dabrafenib/ trametinib, vemurafenib	3 months; BRAF/ MEK inhibitors held for 2–3 days before/after SRS	Not reported	Distant brain control, local control, progression-free survival, overall survival, neurotoxicity	Kruskal–Wallis, Pearson's Chi-squared, Fisher's exact tests. Kaplan Meier and log-rank tests. Cox prop hazards for hazard ratios.	LOW: small, retrospective cohort study. No downgrade required.
Diao et al. (2018)	72	310	Single-fraction SRS Elekta Perfexion Gamma Knife(s)	Anti-CTLA4 = ipilimumab	4 weeks	8.3	Local failure, treatment-related imaging changes, tumor, and edema volumes, neurotoxicity	Kruskal–Wallis, Pearson’s Chi-squared, Fisher's exact tests. Kaplan Meier and Cox proportional hazards for hazard ratios.	LOW: small, retrospective cohort study. No downgrade required.
Diao et al. (2018)	91	256	SRS Perfexion Gamma Knife	Anti-CTLA4 = ipilimumab	4 weeks (peri = 4 wk–3 mo)	7.6	Distant brain failure, local failure, failure-free survival, overall survival, neurotoxicity	Kruskal–Wallis, Pearson’s Chi-squared, Fisher's exact tests. Kaplan Meier and Cox proportional hazards for hazard ratios.	LOW: small, retrospective cohort study. No downgrade required.
Yusuf et al. (2017)	51	167	CyberKnife/Varian Trilogy LINAC	Anti-PD1/anti-CTLA4 = ipilimumab/ pembrolizumab	4 weeks (peri = 4 wk–3 mo)	5.8	Distant brain failure, local failure, percent lesion regression, overall survival, neurotoxicity	Kruskal–Wallis, Pearson's Chi-squared, Fisher's exact tests. Kaplan Meier and Cox proportional hazards for hazard ratios.	LOW: small, retrospective cohort study. No downgrade required.

Results of Concurrent Immunotherapy and SRS

**Figure 2:**
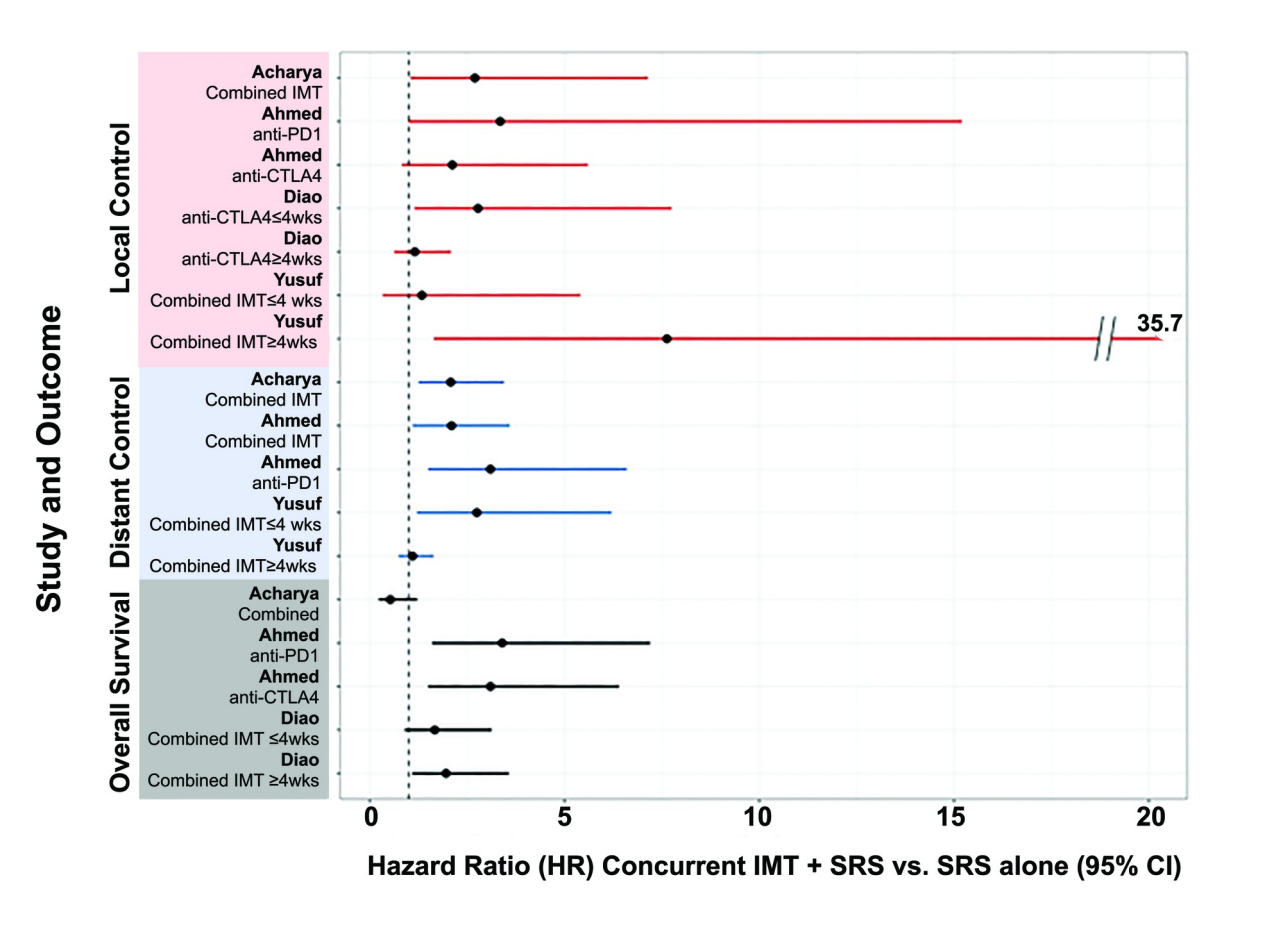
Efficacy of concurrent immunotherapy and SRS for melanoma brain metastases IMT, immunotherapy; CTLA4, cytotoxic T-lymphocyte-associated protein 4; PD1, programmed cell death protein 1; wks, weeks; SRS, stereotactic radiosurgery

Concurrent therapy was generally favored for all reported outcomes. Possibly, the largest effect was on distant brain control combining IMT+SRS (HRs ≥2 for distant control in all but one case; Figure 2). Local control favored concurrent therapy, but this trend was obscured by unexpected differences in HRs for similar patient populations (Figure 2) [9,11]. Overall survival with concurrent IMT+SRS was also improved. Only Acharya et al. found a statistically insignificant decrease in HR for overall survival in patients receiving concurrent IMT and SRS (Figure 2) [7]. There was no significant difference in toxicities between the SRS only and the SRS + IMT treatment groups (Table 2).

**Table 2:** Studies considering treatment efficacy of SRS and immunotherapy SRS, stereotactic radiosurgery; HR, Hazard ratio; NS; not significant; NR, not reported; MBM, melanoma brain metastases; PD-1, programmed cell death protein 1; CTLA4, cytotoxic T-lymphocyte-associated protein 4; IMT, immunotherapy * Converted from failure to control; **chemotherapy controlled; ***compared with immunotherapy before SRS [7-11]

Study	Overall Survival	Local Control	Distant Control	Toxicities	Comparator
Acharya et al. (2017)	0.53 (0.23–1.22) p = NS	2.70 (1.05–7.14) p=0.04*	2.08 (1.25–3.45) p=0.003*	Toxicities were noted in all groups; no differences	SRS alone; controlled for # MBM and steroid use
Ahmed et al. (2016)	Anti-PD-1 3.4 (1.6–7.2) p = 0.0009; Anti-CTLA4 3.1 (1.5–6.4) p = 0.002 **	Anti-PD-1 3.35 (0.99–15.2) p=0.051; Anti-CTLA4 2.12 (0.82–5.6) p=0.12 **	Anti-PD1 3.1 (1.5–6.6) p=0.001; combined IMT 2.1 (1.1–3.6) p=0.02 **	Toxicities were noted in all groups; no differences	SRS alone; chemotherapy controlled
Diao et al. (2018)	NR	Timing ≤4 weeks 2.78 (1.15–7.75);* timing ≥4 weeks 1.16 (0.63–2.09)*	NR	Any lesion hemorrhage HR = 2.13 (0.987–4.72)*	Anti-CTLA4 vs SRS alone
Diao et al. (2018)	Timing ≤4 weeks 1.67 (0.90–3.13)*; timing ≥4 weeks 1.96 (1.09–3.57)* p=0.02;	NR	NR	Toxicities were noted in all groups; no differences	Anti-CTLA4 vs SRS alone
Yusuf et al. (2017)	Median peri-SRS = 7.4 mo; SRS alone = 7.1 mo; p=0.212	Concurrent 1.34 (0.33–5.41)*; peri 7.63 (1.64–35.7)*	Concurrent 2.75 (1.21–6.21)*; peri 1.10 (0.74–1.64)*	Toxicities were noted in all groups; no differences	SRS alone

**Figure 3:**
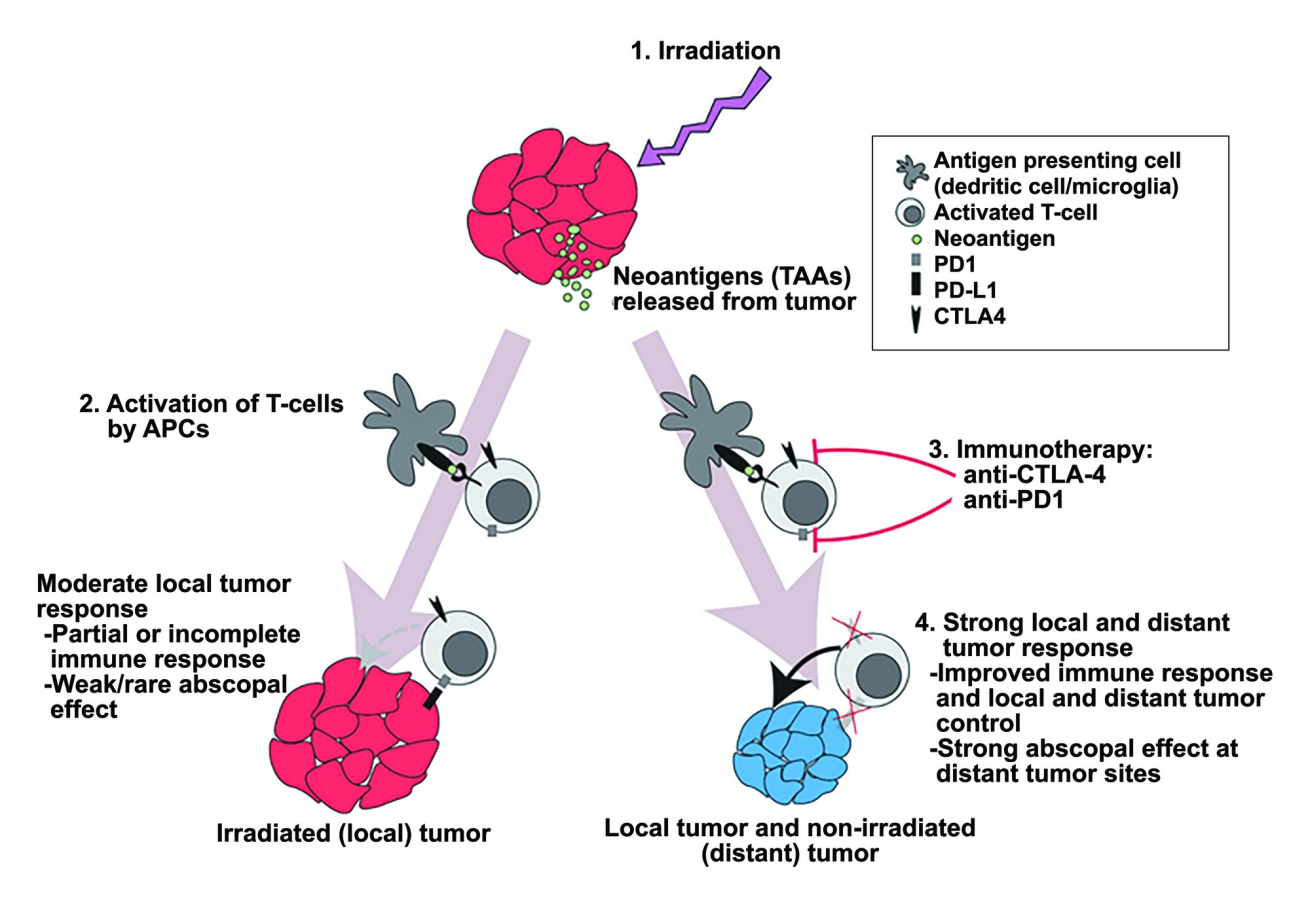
Model of increased abscopal response in combination immunotherapy and radiosurgery 1) Irradiation of tumor causes the release of TAAs. 2) Cytotoxic T-cell activation by APCs. 3) Immunotherapy facilitates T-cell activation (anti-CTLA4) and prevents immune checkpoint activation (anti-PD1). 4) Increased abscopal effect and local tumor responses. TAA, tumor-associated neoantigen; APC, antigen-presenting cell; CTLA4, cytotoxic T-lymphocyte-associated protein 4; PD1, programmed cell death protein 1; PD-L1, programmed death-ligand 1; TT, targeted therapy; SRS, stereotactic radiosurgery; BRAFi, BRAF inhibitor; MEKi, MEK inhibitor. © Department of Neurosurgery, University of Utah

Regression or control of lesions outside of the initial radiation field, called the abscopal effect, may drive favorable outcomes for distant brain tumors in patients treated with concurrent IMT+SRS [12-14]. The abscopal effect is an immune system-mediated effect that requires T-cell effector function on tumor-associated neoantigens (TAAs) and immune-enhancing cytokine release in the tumor microenvironment. This may synergize with checkpoint inhibitors, releasing the brakes on antitumor immunity (Figure 3) [15-17]. The reactivation of innate immune-sensing and interferon responses in tumor cells, which is critical for antitumor immunity, is attributed to the expression of endogenous retroviral sequences and other genomic ‘dark matter’ after epigenetic therapies [18,19-21]. Combining epigenetic, stereotactic radiosurgical, and immune-checkpoint therapies may hold great promise for patients with melanoma brain metastasis.

Results of BRAFi/MEKi and Stereotactic Radiosurgery

**Figure 4:**
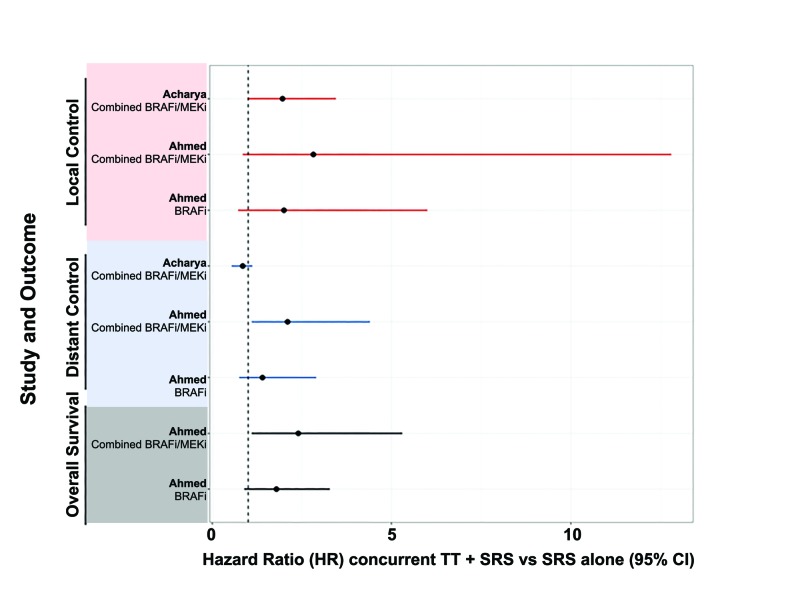
Efficacy of concurrent targeted therapy and SRS for melanoma brain metastases BRAFi, BRAF inhibitor therapy; MEKi, MEK inhibitor therapy; TT, targeted therapy; SRS, stereotactic radiosurgery

Inhibitors of the MAP-kinase pathway have become a mainstay of melanoma treatment, particularly in the setting of activating BRAF mutations. Importantly, patients treated with targeted therapies after molecular diagnosis have significantly better progression-free and overall survival than patients treated with dacarbazine or placebo [22-25]. There is little information regarding the optimal timing of targeted therapy dosage relative to SRS, but many oncological teams withhold targeted therapies for 3-5 days surrounding SRS treatment. Studies that report the efficacy of SRS and targeted therapies are summarized in Table 4, including reason for exclusion from our systematic review [26-30]. Improved brain tumor control and overall survival evident in concurrent or post-SRS (Figure 4; Table 3) BRAFi/MEKi may reflect increased accessibility of these therapies to the brain tumor environment after SRS, yet concurrent vemurafenib or dabrafenib and SRS has been reported to increase risk for radiation necrosis and Grade ≥3 adverse events, especially skin toxicities [30-32]. Of interest, there were no significant differences in toxicities in patients receiving concurrent BRAFi/MEKi+SRS versus SRS alone, indicating that a small pause directly surrounding SRS may be sufficient to ensure patient safety (Table 3).

**Table 3:** Studies considering treatment efficacy of SRS and targeted therapy SRS, stereotactic radiosurgery; NR, not reported in study outcomes; BRAFi, BRAF inhibitor; MEKi, MEK inhibitor *Converted from failure to control; **Chemotherapy controlled [7-11]

Study	Overall Survival	Local Control	Distant Control	Toxicities	Comparator
Acharya et al. (2017)	NR	1.96 (0.99–3.45)* p=0.054	0.85 (0.54–1.12)*; BRAFi/MEKi vs BRAFi alone p=0.011	Toxicities noted in all group; no differences	SRS alone with multiple subgroup analyses
Ahmed et al. (2016)	BRAFi/MEKi 2.4 (1.1–5.3) p = 0.02; BRAFi 1.79 (0.89–3.28) **	BRAFi/MiEKi 2.82 (0.85–12.8) p=0.09; BRAFi 2 (0.72–6.0) p = 0.18 **	BRAFi/MEKi 2.1 (1.1–4.4) p = 0.03; BRAFi alone 1.4 (0.75–2.9) p = 0.27	Toxicities noted in all group; no differences	SRS alone; chemotherapy controlled
Diao et al. (2018)	NR	NR	NR	NR	NR
Diao et al. (2018)	NR	NR	NR	NR	NR
Yusuf et al. (2017)	NR	NR	NR	NR	NR

**Table 4:** Excluded studies considering SRS + BRAFi/MEKi therapies SRS, stereotactic radiosurgery; LC, local control; OS, overall survival; WBRT, whole-brain radiation therapy; HR, hazard ratio [27-30,33]

Study	Outcome	Reason for Exclusion
Kotecha et al., (2017)	Improved LC, OS in concurrent SRS	High number (>30%) WBRT
Mastorakos et al., (2019)	Improved OS initiating inhibitor after SRS	No timing details, no HRs/statistics provided
Wolf et al., (2016)	Improved OS in concurrent or after SRS strategies	No timing details, no HRs/statistics provided
Xu et al., (2017)	Improved LC with any BRAFi + SRS	Small sample size, heterogeneous timing. No HRs/statistics provided
Hecht et al., (2018)	Improved OS in an interrupted therapy group	High number (>30%) WBRT

## Conclusions

Five studies in the literature explore concurrent timing of stereotactic radiosurgery and immunotherapy or targeted therapies for the treatment of intracranial metastatic melanomas. Additional temporally specific studies are needed, but more vital is the need for well-designed prospective trials, several of which are under way [NCT02085070; NCT02374242; NCT02460068; NCT02320058]. Future studies should report outcomes based on a four-week window (concurrent SRS) or a three-month window (peri-SRS).
